# Supplementary material for: Transcriptome Analysis Reveals Vimentin-Induced Disruption of Cell–Cell Associations Augments Breast Cancer Cell Migration
Source: Cells. 2022 Dec 13;11(24):4035. doi: 10.3390/cells11244035 (PMC9776984; doi:10.3390/cells11244035)
Supplement: Supplementary file 1 [file cells-11-04035-s001.zip › DEGs and enrichment analysis.zip!/DEGs and enrichment analysis/WT_VIMvsVector KEGG/UP/WT_VIMvsVector_UP.pathway_map.zip!/WT_VIMvsVector_UP.pathway_map/src/hsa04080.html]

hsa04080


- hsa:2563

- Up regulated genes

ENSG00000187730(3.1977132561098)

- hsa:53637

- Up regulated genes

ENSG00000180739(4.10779050911213)

- hsa:5031

- Up regulated genes

ENSG00000171631(2.06649655266787)

- hsa:4886

- Up regulated genes

ENSG00000164128(3.01346113167213)

- hsa:1128

- Up regulated genes

ENSG00000168539(2.56985566842982)

Close
